# Trends and Limitations in the Assessment of the Contractile Properties of Human Induced Pluripotent Stem Cell-Derived Cardiomyocytes From Patients With Dilated Cardiomyopathy

**DOI:** 10.3389/fcvm.2020.00154

**Published:** 2020-09-03

**Authors:** Masamichi Ito, Seitaro Nomura, Hiroyuki Morita, Issei Komuro

**Affiliations:** Department of Cardiovascular Medicine, Graduate School of Medicine, The University of Tokyo, Tokyo, Japan

**Keywords:** iPS cell-derived cardiomyocytes, dilated cardiomyopathy, contractile property, tissue engineering, disease modeling, high-throughput screening

## Abstract

The application of human induced pluripotent stem cell-derived cardiomyocytes (hiPSCMs) from patients is expected in disease modeling and drug screening *in vitro*. Dilated cardiomyopathy (DCM) is an intractable disease characterized by the impairment of systolic function and leads to severe heart failure. A number of researchers have focused on disease modeling of DCM and reproduced its pathologic phenotypes in hiPSCMs, but a robust method to evaluate the contractile properties of cardiomyocytes *in vitro* has not been standardized. In addition, it is unknown whether the throughput of measurements and analyses could be increased sufficiently for compound screening. Here, we reviewed the articles in which the contractile abnormalities of DCM hiPSCMs were recapitulated and assessed the trends and problems in sample preparation and evaluation. We found that single-cell level analysis was ineffective in some cases, and a tissue engineering approach has become dominant recently because of its increased efficiency in reproducing impaired contractility. We also examined two commercially available automated measurement devices with moderate throughput for motion analysis using two-dimensional hiPSCM sheets composed of originally established DCM hiPSCMs. As a result, both of the tested devices, an impedance analyzer and a video image-based cell motion analyzer, were not effective in detecting the expected reduction of contractility in the DCM clone. These findings collectively suggest that a tissue engineering approach could expand the potential of disease modeling with hiPSCMs, and so far, appropriate methods for *in vitro* force measurement with sufficient throughput, but without sacrificing physiological fidelity, are awaited.

## Introduction

In the field of cardiovascular medicine, studies on myocardial tissue from patients have been quite limited because adult cardiomyocytes do not proliferate and it is technically difficult to continually culture harvested cardiomyocytes *in vitro*. Since human induced pluripotent cells (hiPSCs) were established in 2007, it has been possible to perform experiments with human cardiomyocytes derived from patients with known gene mutations and clinical courses. Their potential for clinical application has been explored rigorously, not only as a source of regenerative medicine but also as a tool for disease modeling. Especially, early-onset diseases caused by germline gene mutations are assumed to be a good candidate for disease modeling with hiPSCs. As cardiomyopathy is generally caused by gene mutations, it is highly expected that hiPSC-derived cardiomyocytes (hiPSCMs) from patients with cardiomyopathy could reproduce the pathogenic process and be used as a tool for drug screening.

Dilated cardiomyopathy (DCM) is characterized by dilation of the heart chamber and decreased systolic function. Patients with DCM have a poor prognosis because of accompanying complications such as severe heart failure and fatal arrhythmia. In Japan, more than 27,000 patients are diagnosed with DCM and it is the most common cause (>66%) of heart transplantation (1). Today, mutations in more than 50 genes, such as sarcomeric proteins, ion channels, and molecules of the sarcoplasmic reticulum, have been identified as pathogenic for DCM (2). However, the precise disease mechanism is not understood fully, and therefore a drug therapy specifically targeting DCM has not been established.

Recently, a number of groups have reported studies focusing on the disease mechanism of DCM using patient-specific hiPSCMs. Differences between DCM and control hiPSCM clones have been examined widely from various aspects, such as cellular morphology, gene expression, intracellular signaling, and physiological phenotypes including electrical activity and calcium handling. For example, Lee et al. reported the abnormal activation of platelet-derived growth factor pathways in lamin A/C (*LMNA*)-mutant DCM hiPSCMs and showed that a platelet-derived growth factor receptor inhibitor rescued arrhythmic activity, which provided a potentially new paradigm for the management of DCM-related arrhythmia (3). However, as the clinical diagnosis of DCM is confirmed by the presence of systolic abnormalities, the evaluation, and rescue of contractile abnormalities are the most essential factors in the management of DCM. In order to perform phenotypic screening of therapeutic compounds for DCM, it is necessary to develop *in vitro* models that reproduce the contractile abnormalities, as well as methods for assessing the contractile properties precisely.

The methods for assessing cardiomyocyte contractility *in vitro* were limited until recently due to technical issues. However, considerable progress has been achieved in sample preparation and functional analysis of hiPSCMs in the last decade, which enabled the detection of abnormalities in DCM clones. We should identify which features we can and cannot recapitulate with hiPSCMs using the currently available systems and find a way to overcome their limitations in order to apply these advances to drug development. In order to conduct compound screening, scaling up the systems to a high-throughput platform and automating measurements and analytic processes are also important issues.

In this article, we reviewed studies of disease modeling using DCM hiPSCMs, especially focusing on those in which the contractile properties of cardiomyocytes were measured *in vitro* and summarized the trends of the analytic methods and approaches used for sample preparation. Furthermore, to assess the possibility of high-throughput analyses, we performed contraction assays with two commercially available automated analyzers using our own DCM-specific hiPSCMs.

## Review of the Literature: Current Status of *In vitro* Contractile Analysis

To date, a number of articles have been published trying to create a disease model using hiPSCMs established from patients with DCM. Here, we identified the articles that successfully evaluated the contractile properties of hiPSCMs and reviewed the trends, ingenuities, and limitations in sample preparation and analytic methodology. A summary of the findings is shown in Table 1.

**Table 1:** Overview of the studies analyzing contractile properties of hiPSCMs from DCM patients.

**Author**	**Mutation**	**Sample**	**Timing of analysis**	**Purification of cardiomyocytes**	**Component of engineered tissue**	**Measurement method**	**Measured index**	**Isogenic control line**	**Tested treatment**	**Outcome (disease modeling of DCM and its rescue)**	**Disadvantage in the way of contractility analysis**	**Year**
Sun et al.	*TNNT2* p.R173W	Single cell	Day 18–48	None	N.A.	Atomic force microscopy with a silicon nitride cantilever	Contractile force (deflection × spring constant)	Not included	Gene induction of SERCA2a (adenovirus vector, MOI100, 48 h)	•Successful [contraction force decreased in the DCM clone]•Induction of SERCA2a rescued the contraction abnormality of the DCM clone	Low throughputs Required considerable numbers of measurement (100–400 beats/cell)	2012 (4)
Wu et al.	*TNNT2* p.R173W	Single cell	Day 60	Glucose-free medium for several rounds (purity >90%)	N.A.	Traction force microscopy	Tangential stress Maximum contract rate	Not included	ISO (1 μM, 30 min) Milrinone (PDE3A inhibitor, 10 μM, 15 min) NAY-60–7550 (PDE2A inhibitor, 100 nM, 15 min)	•Successful [peak tangential stress and maximum contract rate were decreased in DCM clone] [inotropic augmentation of contractility by ISO was impaired in DCM clone] •PDE2A/3A inhibitors rescued the impaired beta-adrenergic signaling of DCM clone	Low throughputs	2015 (5)
Broughton et al.	*TNNT2* p.R173W	Single cell/cultured on 100 kPa PDMS substrate	Stocked at day18–48; analyzed 1 week or 2 weeks after thawing	None	N.A.	Line scan of live cell video recording	Maximum shortening	Not included	Omecamtiv mecarbil (sarcomere activator; 200 nM, 500 nM, 1 week)	•Partially successful (in one clone, at early stage of culture) [contractile force; control > DCM] •Omecamtiv mecarbil increased the contractility of the DCM clone	The phenotype was not reproduced in another clone Longer culture resulted in decreased difference of the contractility	2016 (6)
Hinson et al.	*TTN* truncation (A band) (p.A22352fs^+/−^, p.P22582fs^+/−^), missense mutation(p.W976R^+/−^)	Single cell	Day 30–40	Metabolic selection(purity >90%)	N.A.	➀Microarray post-detector (detection of the displacement of fluorescent images of micro-post-deflection)	2D contractile traction force, strain energy	Not included	N.A.	•Failed	The analysis requires specialized device, technique and efforts to fabricate the microtissue	2015 (7)
		➁Engineered tissue			hiPSCM Human mesenchymal stem cells (7%) Collagen 1 Fibrinogen	➁Twitching force calculation based on the displacement of fluorescent micro-beads embedded in the tissue	Twitch force	Included; the mutations were introduced into control iPS cell line using CRISPR/Cas9	VEGF (50 ng/mL, 4 days)	•Successful [contraction force decreased in the DCM clone] •VEGF improved force production of the DCM engineered tissue		
Yang et al.	*MYH7* p.E848G	Single cell	Day 30, 50	None(purity >60%)	N.A.	Optical video microscopy (IonOptix); detection of cell edge shortening	Cell edge deformation; fractional shortening	Included; the mutant protein was induced in MYH7-null cardiomyocytes	N.A.	•Partially successful [fractional shortening was smaller in DCM clones at day 50, not at day 30]	Long term culture was required	2018 (8)
		➁Engineered tissue	Day 28–35		hiPSCM Marrow stromal cells (16%) Collagen 1 Mouse basement membrane extract	➁Force transducer put in the tissue	Active force, passive force, contractile kinetics	Not included		•Successful [contractile function was impaired in the DCM clone]	•Specialized device, technique, and efforts to fabricate the microtissue was required •Number of the experiments was limited	
Zaunbrecher et al.	*TTN* homozygous truncation mutation (A band, Z band)	Single cell	Day 35–40	Metabolic selection	N.A.	➀PDMS micro-post-array	Twitch force, passive force	Included; truncation mutants were induced in wild type iPS cell line using CRISPR/Cas9	N.A.	•Successful [slight reduction in twitch force was observed in TTN- Z^−/−^ DCM clones]	•Considerable number of measurements (*n* > 100) was performed •Data showed high variability	2019 (9)
		➁Engineered tissue	7 weeks		hiPSCM Marrow stromal cells (10%) Fibrin	➁Light microscopy; force calculated based on the deflection of the posts	Twitch force, active tension			•Successful [remarkable reduction in twitch force was observed in TTN- Z^−/−^ DCM clones]	Specialized device, technique and efforts to fabricate the microtissue was required	
Streckfuss-Bomeke et al.	*RBM20* p.S365A	Engineered tissue	Day 60	Metabolic selection	hiPSCM (purity 65–80%) Collagen 1	Force transducer active force; under isometric condition passive force; under 1.5 Hz stimulation	Active force generation Passive force	Not included	N.A.	•Successful [contraction force and passive stress were reduced in the DCM clone]	Specialized device, technique and efforts to fabricate the microtissue was required	2017 (10)
Ceholski et al.	*PLN* p.R9C	Engineered tissue	6 weeks	None	hiPSCM Collagen	Real-time non-invasive optical tracking of integrated endposts	Developed force, contractile kinetics	Included; truncation mutants were induced in wild type iPS cell line using CRISPR/Cas9	N.A.	•Successful [slight reduction was observed in developed force of the DCM clone] [attenuated chronotropic and lusitropic response to beta adrenergic stimulation was found in DCM]	Low throughputs	2018 (11)

*Red, samples prepared in a tissue engineering approach*.

*Blue, parameters directly measured from an attachment on the cell*.

*Green, parameters calculated based on the displacement of spots integrated with the cell*.

In the first period of this research field, contractile properties were analyzed at the single-cell level using cardiomyocytes derived from a patient with a mutation in troponin T2, cardiac type (*TNNT2*; p.R173W), which constitutes a sarcomeric thin filament and is one of the major causative genes of DCM. Sun et al. (4) evaluated the contractile force of hiPSCMs using atomic force microscopy and revealed the impaired contractility of the DCM clone. Atomic force microscopy enables quantification of motion dynamics by measuring slight deformations of the cell surface by a cantilever attached to a beating cell. Wu et al. (5) also analyzed DCM hiPSCMs with the same mutation. In their study, the functional properties of hiPSCMs were assessed using traction force microscopy: hiPSCMs were seeded on hydrogel-embedded fluorescent beads and contractility was calculated based on deviations of the beads. The authors revealed that the contractility of DCM hiPSCMs was significantly reduced compared with control clones. It was promising that the contractile abnormality of hiPSCMs was commonly observed using two different modalities in the same cell model. However, cell-to-cell variability of the measurements was rather high, and the measurements had to be repeated a considerable number of times (100–400 beats). The amount of labor required for image acquisition and calculation was also a bottleneck for its application to high-throughput screening.

Broughton et al. (6) also assessed the contraction of *TNNT2-*mutant hiPSCMs, and their findings raised several questions regarding single-cell contraction analysis *in vitro*. They recorded live-cell video images of beating hiPSCMs and performed kymograph analysis of line scans to calculate cellular shortening and its velocity. They showed that cell shortening was decreased in the mutant clone compared with the normal clone, but the difference between both clones became smaller after a longer period of culture. In addition, the difference did not reach statistical significance in another pair of mutant and normal clones. They also showed that contractility varied based on the stiffness of the substrate the cells were seeded on. Their results suggest that many of the culture and measurement conditions could affect contractile status *in vitro*, which resulted in high variability and low reproducibility.

After the limitations of single-cell level analysis became apparent, several researchers produced engineered heart tissues containing cardiomyocytes to enhance the physiological fidelity of the material. Among the earliest studies using this concept, Hinson et al. (7) analyzed hiPSCMs with mutations in titin (*TTN*): two A-band frameshift mutations (p.A22352fs^+/−^, p.P22582fs^+/−^) and a missense mutation in the Z/I junction (p.W976R ^+/−^). *TTN* is the most frequently mutated gene in DCM and accounts for approximately 20% of patients (2). Interestingly, although the difference of contractile energy was not obvious between normal and DCM clones when measured at the single-cell level, a remarkable reduction of twitch force was observed in engineered heart tissues produced using DCM hiPSCMs, indicating that this tissue engineering approach was quite effective in disease modeling of DCM. Some studies followed this milestone and performed both single-cell and heart tissue contraction analyses. Yang et al. (8) analyzed the samples from a familial DCM cohort with a mutation in myosin heavy chain 7. They also demonstrated the weakened contractile properties of DCM engineered heart tissue. On the other hand, abnormalities in contractile function were evident at the single-cell level in day 50 hiPSCMs, but not in day 30 hiPSCMs. Similarly, Zaunbrecher et al. (9) induced homozygous truncations in the Z-disk and A-band of *TTN* and generated *TTN* knock-out hiPSCMs to analyze the impact of *TTN* mutation on sarcomere formation and function. They evaluated force production in hiPSCMs with engineered heart tissue and single-cell models and found that the reduction of twitch force was prominent in the mutant clones. The degree of force reduction was more outstanding in the analysis using engineered heart tissue. Several other papers have reported disease modeling of DCM hiPSCMs with different gene mutations (10, 11). Although the methods for sample preparation and force detection were slightly different, the use of engineered heart tissue has become a mainstream approach for the assessment of contractile abnormalities. As discussed later, such an approach may be helpful in highlighting disease-specific abnormalities by improving the maturity of hiPSCMs as well as their physiological validity in cellular alignment and connections. Further progress in tissue engineering will contribute to more precise disease modeling of cardiomyopathy; however, its direct application to high-throughput drug screening is not yet practical, as it requires special devices, techniques, and labor to prepare the samples.

In addition, a cross-sectional review of the literature revealed that there were numerous discrepancies in the methods used, other than the contractile assay, which could lead to variation in outcome among studies. In the course of analyzing the functional phenotype, there are a number of considerations, including the clone-to-clone and batch-to-batch variances of the cells being prepared, the method for sample preparation, the day of analysis after the start of differentiation, and the conditions of drug administration. In the current review, for example, the non-cardiomyocyte cells (e.g., marrow stromal cells and mesenchymal stem cells) and extracellular matrix (e.g., fibrin and collagen) used in the construction of engineered heart tissues were found to be diverse. The day of analysis was also diverse (i.e., from day 30 to 60). Furthermore, most drug administration experiments were conducted at only one concentration for a certain period, making it difficult to evaluate the reproducibility of the drug effect. These differences can result in high inter-experimental variability and low robustness of studies in this field. The development of standardized protocols for materials and methods using hiPSCMs is desired.

Isogenic control lines have been used frequently in recent years to better define the significance of genetic mutations and to clarify the causal relationship between these mutations and disease-specific phenotypes. Isogenic control lines are created by genome editing techniques, such as CRISPR/Cas9, and their genetic background, other than the responsible mutation(s), is identical to the original clone. We reviewed whether isogenic control lines were prepared in each study. As a result, they have been included more frequently since 2018. It seems to be one of the factors determining the success of reproducing the disease phenotype of various mutations, and it appears to have become standard to use isogenic control lines in recent studies. In the reviewed papers, all isogenic control lines were created by knocking in the responsible mutation to the healthy strain, and there were no studies that corrected the responsible mutation in the disease line. In the future, bidirectional comparisons, of both inducing and repairing the mutation, may be helpful to further define the relationship between mutation and phenotype.

## Original data: High-Throughput Analysis of hiPSCM contractile Properties

After reviewing recent articles, we realized that the currently popular methods to evaluate the contractile function of cardiomyocytes *in vitro* had limitations in their application to high-throughput screening; the single-cell approach lacks physiological fidelity and needs repeated numbers of tests owing to variance, while the tissue engineering approach requires considerable time and labor to construct the samples. In order to establish a drug screening system based on the abnormal phenotypes of patient-specific hiPSCMs, we tried to measure the contractile properties of hiPSCMs in a two-dimensional (2D) cell sheet-based manner using commercially available automated analytic devices, aiming to maintain physiological relevance and experimental throughput. We assessed the performance of two devices that enable non-labeled analysis of hiPSCM sheets: the xCELLigence RTCA CardioECR System® (ACEA Bioscience, Inc., San Diego, CA) (12) and the SI8000 Cell Motion Imaging System® (Sony, Tokyo, Japan) (13). In these experiments, we established hiPSC lines from a DCM patient with a mutation in *LMNA*, which is associated with poor prognosis.

## Materials and Methods

### Patient Recruitment

**Figure 1:**
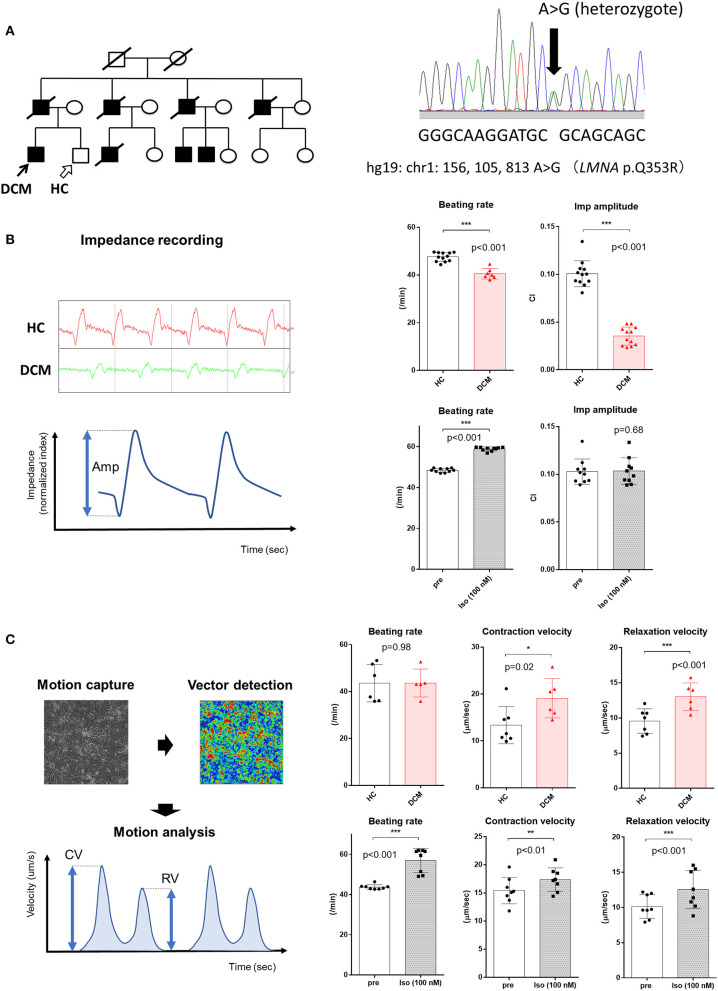
Contractile properties of *LMNA*-mutant DCM hiPSCMs assessed in the 2D sheet approach. **(A)** (Left) Pedigree of a familial DCM cohort with a heterozygous mutation in *LMNA*. Black arrow indicates the DCM patient; white arrow indicates the healthy sibling who served as a healthy control (HC). (Right) Sanger sequencing of DNA from the patient indicating a heterozygous mutation in *LMNA*. **(B)** (Left) Representative images of impedance recording obtained from xCELLigence CardioECR. Red, recording from the HC sample; green, recording from the DCM patient sample. Amp, amplitude. (Upper Right) Comparison of beating rate and impedance amplitude obtained from HC and DCM hiPSCM sheets (HC, *n* = 12; DCM, *n* = 13). CI, cellular impedance. (Lower Right) Comparison of beating rate and impedance amplitude of HC hiPSCM sheets before and after treatment with 100 nM isoproterenol (*n* = 10, respectively). **(C)** (Left) Conceptual image of the Cell Motion Imaging System and parameters calculated by the device. CV, contraction velocity; RV, relaxation velocity. (Upper Right) Comparison of beating rate, CV, and RV obtained from HC and DCM hiPSCM sheets (HC, *n* = 7; DCM, *n* = 6). (Upper Left) Comparison of beating rate, CV, and RV of HC hiPSCM sheets before and after treatment with 100 nM isoproterenol (*n* = 8, respectively). *p < 0.05; **p < 0.01; ***p < 0.001.

A 43-year-old male DCM patient was enrolled for this study. Multiple members of his family had a history of DCM and sudden cardiac death. The patient initially presented with a complete atrioventricular block and underwent permanent pacemaker implantation at the age of 31 years. At that time, he was also diagnosed as DCM by transthoracic echocardiography and heart biopsy. Five years later, a cardioverter defibrillator was implanted due to sustained ventricular tachycardia. The following year, a left ventricular assist device was implanted because of refractory heart failure. Then, he received a heart transplant at the age of 39 years. A heterozygous missense mutation in *LMNA* (p.Q353R) was revealed by panel sequencing of candidate cardiomyopathy genes (Figure 1A). A healthy male sibling of the patient was also enrolled as a healthy control (HC) subject. The study was approved by the Institutional Review Board of the University of Tokyo Hospital (11044). Written informed consent was obtained from the participants for the publication of any potentially identifiable images or data included in this article.

The other methods are described in the Supplementary Materials.

### Results

hiPSCMs were produced from a male patient with an *LMNA* mutation and his healthy sibling (Figure 1A, Supplementary Figure 1). The CardioECR system enables the analysis of the contractile activity of hiPSCM sheets that are synchronously beating in multiple wells at the same time. We compared the contractile function of hiPSCMs from the HC and DCM patient. The CardioECR system was used to continuously record post-seeding myocardial status, which showed a decrease in well-to-well variability in the first week after seeding (Supplementary Figure 2). The DCM clone showed a slight but significant decrease in the beating rate. We also found that the amplitude of cellular impedance, which corresponds to the magnitude of contractility, of the DCM clone was significantly smaller than that of the HC clone. This result was reasonable considering that DCM hearts harboring an *LMNA* mutation show a progressive impairment of systolic function. We then evaluated the response of the contractility of the control hiPSCMs to the administration of isoproterenol, a beta-adrenergic stimulant. Contrary to expectations, no increase was observed in the amplitude of cellular impedance after the addition of isoproterenol. These results suggest that this system partially reproduced the functional abnormalities of DCM; however, it would not be suitable for analysis of the pharmacological response including beta-adrenergic receptor signaling (Figure 1B, Supplementary Figure 3).

Next, we analyzed the contraction of hiPSCM sheets using the Cell Motion Imaging System with the same schedule. This device automatically records videos from multiple points of a plate in a single experiment and calculates the contractile properties of beating cells, such as contraction velocity (CV) and relaxation velocity (RV). Unexpectedly, CV and RV were significantly higher in DCM hiPSCMs compared with HC hiPSCMs. Assuming that the conditions for attachment and cellular stiffness were similar between both hiPSCM clones, it is difficult to give a rational interpretation of these results, considering that DCM is associated with decreased contractile and relaxation functions. On the other hand, when isoproterenol was added to the hiPSCMs, CV and RV were both increased significantly, which suggested that the positive inotropic and lusitropic effects of the beta-adrenergic stimulant could be assessed using this system (Figure 1C).

Collectively, each of these two systems could be applied for moderate-throughput experiments because of the simplicity of data collection. However, the results did not necessarily reproduce the properties of cardiomyocytes in physiologically and pharmacologically expected manners; therefore, it is considered to be difficult to adopt these systems for phenotypic screening of DCM using disease-specific hiPSCMs.

## Discussion

### Principle of Force Detection and Major Methods for Measuring Contractility

In the reviewed articles, the fundamental principle for evaluating contractile force *in vitro* was similar in every experiment; it was assumed that contractile force correlates with surrogate physical metrics such as deformation of cell shape or scaffold caused by the beating of cardiomyocytes. However, diverse methods were used to quantitate deformation and to calculate force. According to our review, the principle of deformation determination *in vitro* could be classified into three groups: (1) detection by probes attached directly to the cell surface (highlighted in blue text in Table 1); (2) observation of the dislocation of scaffolds or spots integrated with the cell (highlighted in green text in Table 1); and (3) video image analysis of cell shape (shown in black text in Table 1). Among these methods, only those in group (1) can calculate force directly, and by the methods used in group (2), force is calculated based on the known material properties of the substratum. Only deformation and/or movement of the cell are measured by the methods used in group (3). A representative example for group (1) is atomic force microscopy, which measures cellular deformation and elasticity with a cantilever placed on the cell surface (14). This type of measurement provides direct and physically well-defined metrics; however, probe application requires technical training and the sample can be damaged during analysis. Group (2) was the most popular approach to analyze deformation. For example, a hydrogel containing fluorescent microspheres or regularly spaced microposts on which the cells are seeded is commonly used as an alternative material that mirrors cellular deflection (15). This method can provide high-resolution and physically relevant information on cell motion. However, the labor required for sample fabrication could limit the throughput of analysis. In video image analysis (3), cellular deformation is detected by optical flow analysis of bright microscopic image sequences and the magnitude of a motion vector is quantitated computationally. Compared with the other two groups, video image analysis is easier, less expensive to establish, and applicable to multiple types of samples (16). However, the detected parameter is a surrogate metric and its calculation depends widely on undemonstrated assumptions. In the current review, two studies adopted video image analysis of cardiomyocytes at the single-cell level (6, 8). In both studies, the variance of the results was considerably high depending on analysis timing and culture conditions. Therefore, only partial reproduction of the contractile abnormality in the DCM clone was achieved. We consider that video image-based analysis would not be superior to the other two groups for the identification of disease-specific phenotypes, presumably as it achieves simplicity at the expense of physiological relevance.

If we were able to describe contractile forces in a quantitative fashion, the results would be more objective and reproducible, and the data from each study would be understood in a unified way. As we mentioned above, most of the methods for evaluating contractile force *in vitro* calculate force indirectly from surrogate parameters. Therefore, the precise quantification of force is not guaranteed with certainty. Kijlstra et al. (17) developed a novel video-based method for estimating contractile force based on the deformation distance of the outline of beating cells. They demonstrated the validity of their method by showing that the results were consistent with measurements using traction force microscopy. As described in that paper, verification of a new approach requires a number of specialized instruments, special sample adjustments, and several theoretical assumptions. The application of new methods to disease cell lines or under pharmacological conditions would also need additional detailed verification.

### Advantages of the Tissue Engineering Approach

With regard to sample preparation, the studies of Hinson et al. (7) and Yang et al. (8) were substantially suggestive; they tried head-to-head comparisons of single-cell vs. tissue engineering approaches using the same cardiomyocytes and revealed that the tissue-based measurements were superior in detecting contractile abnormalities in disease clones. We can summarize the key advantages of the tissue engineering approach in two major points. First, it could promote functional and physiological maturation of hiPSCMs. Engineered heart tissues are generally created by mixing cardiomyocytes and other types of cells with extracellular matrix and encapsulating them in a hydrogel. Collagen, fibronectin, and laminin were among the most commonly used extracellular matrices, which could influence the attachment, proliferation, and physiological properties of cardiomyocytes (18). The extracellular matrix and hydrogel also increase substrate stiffness, and previous studies reported that higher stiffness (>10 kPa) induced augmented force generation and reinforced calcium transients in cardiomyocytes (19). The coexistence of non-cardiomyocyte cells, such as fibroblasts and endothelial cells, could also have an impact on maturation through direct contacts and paracrine effects (20). Second, the culture environment more closely mimics the structure of the natural organ. A synthetic scaffold engineered from polymeric materials helped to generate uniaxially aligned and rod-shaped cardiomyocytes with anisotropic sarcomere organization. A designed mold also increased cellular density to a physiologically relevant level, and tissue anchoring achieved the tension-loaded status of cardiomyocytes and enabled physiological isotonic contractions, different from the isometric deformations of dish-attached cells. In summary, engineered heart tissue mirrors the native myocardial niche *in vivo* and facilitates electromechanical maturation toward an adult heart-like phenotype (21). Therefore, there is no doubt that a three-dimensional (3D) tissue engineering approach is a powerful method to model diseases with contractile abnormalities, except for its limitation in scalability.

### Challenges in 2D Sheet-Based Phenotypic Screening

From our results, the reproduction of disease-specific phenotypes seems unfeasible with 2D sheet models. CardioECR is a unique system for cellular contraction analysis that measures the changes of impedance on an electrode. Zhang et al. (12) utilized this device to detect the effect of ion channel blockers on hiPSCMs and assessed their arrhythmogenic properties. In our experiment, the positive inotropic effect of isoproterenol was not reflected in the amplitude of impedance, obviously limiting the usefulness of this approach for drug screening. Consistent with a previous study (22), we could evaluate the effect of isoproterenol precisely with the Cell Motion Imaging System, but the expected differences between the DCM and control clones were not observed. We speculate that the parameters evaluated here were oversimplified to improve convenience in the analytic process, neglecting essential factors that could affect motion generation. Taken together, it is apparently still challenging to apply 2D sheet analysis for drug screening. Improvements in the tissue engineering approach, namely, constructing 3D tissue systems that can reproduce pathophysiology, such as by using sheets or bundles with a minimized number of cells and matrix, could facilitate drug screening with patient-specific hiPSCMs.

## Data Availability Statement

The original contributions presented in the study are included in the article/Supplementary Material, further inquiries can be directed to the corresponding author/s.

## Ethics Statement

The studies involving human participants were reviewed and approved by the Institutional Review Board of the University of Tokyo Hospital (11044). The patients/participants provided their written informed consent to participate in this study.

## Author Contributions

MI reviewed the articles, performed experiments, and wrote the manuscript. SN and HM discussed the data and revised the manuscript. IK supervised the project. All authors contributed to the article and approved the submitted version.

## Conflict of Interest

The authors declare that the research was conducted in the absence of any commercial or financial relationships that could be construed as a potential conflict of interest.

The handling editor declared past co-authorships with author IK.
