# Supplementary material for: Trends and Limitations in the Assessment of the Contractile Properties of Human Induced Pluripotent Stem Cell-Derived Cardiomyocytes From Patients With Dilated Cardiomyopathy
Source: Front Cardiovasc Med. 2020 Sep 3;7:154. doi: 10.3389/fcvm.2020.00154 (PMC7494730; doi:10.3389/fcvm.2020.00154)
Supplement: Supplementary file 1 [file Data_Sheet_1.docx]

Trends and Limitations in the Assessment of the Contractile Properties of Human Induced Pluripotent Stem Cell-derived Cardiomyocytes from Patients with Dilated Cardiomyopathy

**Masamichi Ito^1^, Seitaro Nomura^1^, Hiroyuki Morita^1^, Issei Komuro^1^**

^1^ Department of Cardiovascular Medicine, Graduate School of Medicine, The University of Tokyo, Tokyo, Japan

**Supplementary Methods**

・**Preparation of hiPSCMs**

hiPSC were produced and differentiated into cardiomyocytes as described previously [[1](#_ENREF_1)]. Briefly, hiPSC lines were isolated from the peripheral blood of donors using episomal plasmids. For cardiac differentiation, we adopted a chemically defined protocol. We purified cardiomyocytes according to their specific gravity as described previously [[2](#_ENREF_2)]. Using a machine called an elutriator, the cells were refluxed at various flow rates in an opposite direction to the centrifugal force generated in the rotating chamber, allowing cellular populations with specific sedimentation coefficients to be separated (Fig.1A). Differentiated cells were loaded into an Avanti J-26S XP centrifuge (Beckman Coulter, Brea, CA) equipped with a JE-5.0 rotor (Beckman Coulter) and a 5 mL standard chamber (Beckman Coulter) at various conditions (Fig.S1B). After sorting the cells, we evaluated the purity of cardiomyocytes by immunostaining each fraction. As a result, it was found that a population of highly purified cardiomyocytes with a TNNT2-positive rate of 80% or more can be obtained by collecting the cells distributed in fractions 3 and 4 (Fig.S1C). The cardiomyocytes from these fractions were used in this analysis.

The hiPSCMs were frozen once for storage on day 28. The stocks were then thawed and 2 days later, were passaged for each assay. Contractile analysis was performed at approximately 35 days after differentiation. Analysis was also performed at 15 mins after the addition of 100 nM isoproterenol as a sufficient chronotropic change was confirmed under these conditions (Fig. S3).

**・Measurement of contractile properties with the xCELLigence RTCA CardioECR System**

The xCELLigence RTCA CardioECR System (ACEA Bioscience, Inc., San Diego, CA) was used to measure the impedance of hiPSCMs, which is supposed to correspond to the contractile property of the cells. The E-Plate CardioECR 48 well was coated with 10 μg/mL fibronectin solution. The next day, the fibronectin solution was exchanged with culture medium (Dulbecco’s modified Eagle’s medium containing 10% fetal bovine serum), and the plate was placed in the CardioECR station to perform background measurements prior to cell seeding. hiPSCMs were seeded in each well at a density of 6.0 × 10^5^/cm^2^ (20,000 cells/well). The medium was exchanged every other day. Cell status quality control was performed at 6 days after seeding. Only the wells that met the quality control criteria provided by the supplier were used for the experiment. At day 7, the impedance of the hiPSCMs was obtained for 30 s from each well and analyzed with RTCA CardioECR software.

**・Measurement of contractile properties with the Cell Motion Imaging System**

To evaluate the contractile function of hiPSCMs through a motion vector analysis system, we used the SI8000 Cell Motion Imaging System® (Sony, Tokyo, Japan). This system enables quantitative analysis of the motion of synchronously beating hiPSCMs through video capture. The motion of each detection point was converted into a motion vector. The motion velocity within each region of interest was calculated based on the sum of the vector magnitudes. The maximum contraction velocity is considered to correspond to the contractile function of the myocardium. Similarly, the maximum relaxation velocity is considered to correspond to diastolic function. Video images were taken for 10 s on approximately day 35 of differentiation and each parameter was analyzed using the manufacturer’s software (SI8000 analyzer software).

**・Statistical analysis**

All data analysis was performed using GraphPad Prism 7.04 (GraphPad Software, Inc., San Diego, CA). Data are shown as the mean ± standard deviation. The significance of differences among means was evaluated using Student’s *t*-test or one-way analysis of variance followed by Dunnett’s test. For all analyses, p-values less than 0.05 were considered statistically significant.

**References**

[1] M. Ito, H. Hara, N. Takeda, A.T. Naito, S. Nomura, M. Kondo, Y. Hata, M. Uchiyama, H. Morita, and I. Komuro, Characterization of a small molecule that promotes cell cycle activation of human induced pluripotent stem cell-derived cardiomyocytes. Journal of molecular and cellular cardiology 128 (2019) 90-95.

[2] M. Boerma, C.G. van der Wees, J. Wondergem, A. van der Laarse, M. Persoon, A.A. van Zeeland, and L.H. Mullenders, Separation of neonatal rat ventricular myocytes and non-myocytes by centrifugal elutriation. Pflugers Archiv : European journal of physiology 444 (2002) 452-6.

Supplementary Figures


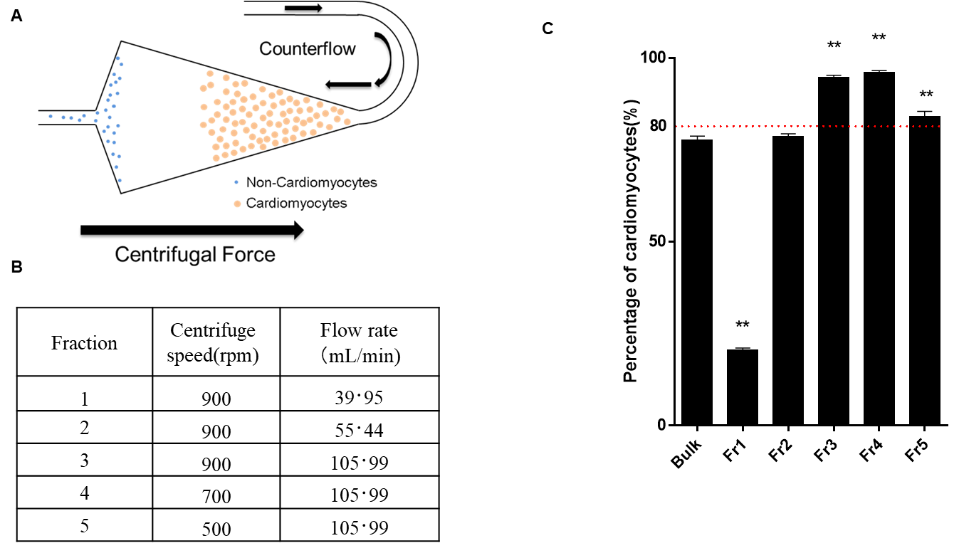


**Supplementary Figure 1. Purification of cardiomyocytes using an elutriation system.**

(A) Schematic depiction of cardiomyocyte enrichment using an elutriation system.

(B) Conditions of centrifugation and counter flow rate for cellular fractionation used for cardiomyocyte enrichment.

(C) Results of immunostaining for TNNT2 in the cells from each fraction (n = 6, **p < 0.01).


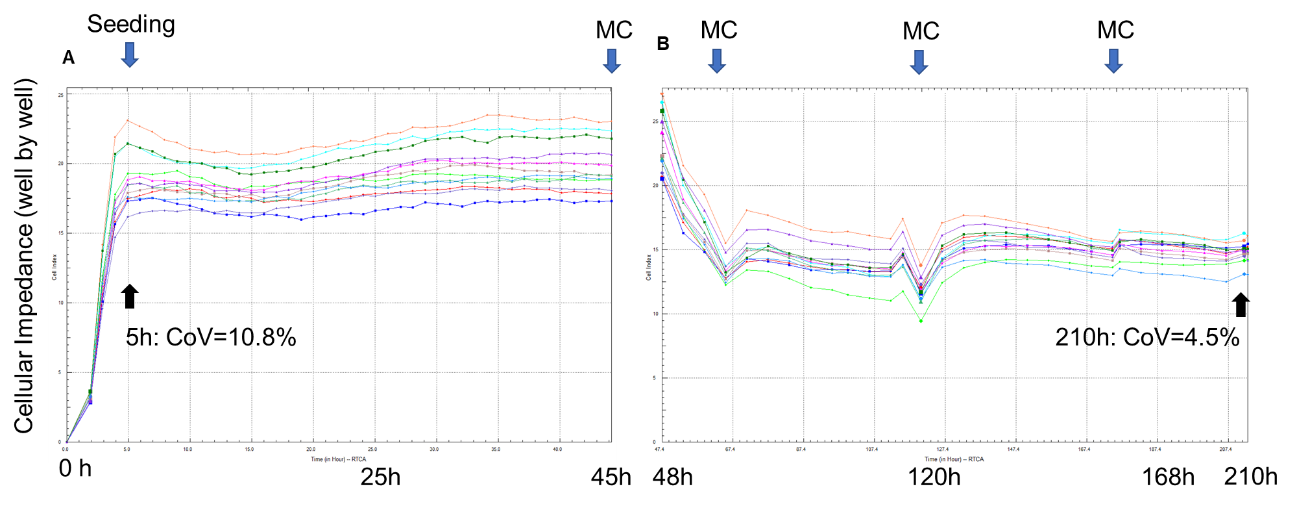


**Supplementary Figure 2. Time course of cellular impedance measured by CardioECR and its well-to-well variance.**

Temporal change of cellular impedance of healthy hiPSCMs from multiple wells (n = 12). Each colored dot and line indicates the impedance measured from a single well. (A) At 0–45 h after seeding, recorded every hour. (B) At 48–210 h after seeding, recorded every 2 h. MC, medium change; CoV, coefficient of variance.

**Supplementary Figure 3. Effect of isoproterenol on the beating rate and cellular impedance of beating monolayer hiPSCMs under several conditions.**

(Upper) Comparison of beating rate (left) and impedance amplitude (right) of HC hiPSCM sheets after administration of isoproterenol (0, 10, and 100 nM). Data from 15 min and 12 h were normalized based on data before treatment (n = 4, respectively). Error bar shows standard deviation of the group. Multiple comparisons by Bonferroni’s method were performed. **p < 0.003, *p < 0.017.

(Lower) Comparison of beating rate (left) and impedance amplitude (right) of DCM hiPSCM sheets after administration of isoproterenol (0, 10, and 100 nM). Data from 15 min and 12 h were normalized based on data before treatment (n = 4, respectively). Error bar shows standard deviation of the group. Multiple comparisons by Bonferroni’s method were performed. **p < 0.003, *p < 0.017.
